# The symbolic-representational construction of COVID-19 in vulnerable population groups

**DOI:** 10.1590/0034-7167-2024-0120

**Published:** 2025-03-17

**Authors:** Denize Cristina de Oliveira, Vanessa Bittencourt Ribeiro, Yndira Ita Machado, Sergio Correa Marques, Jessica Grativol Aguiar Dias de Oliveira, Hellen Polliana Cecílio, Renata Lacerda Marques Stefaisk, Juliana Pereira Domingues

**Affiliations:** I Universidade do Estado do Rio de Janeiro Rio de Janeiro Rio de Janeiro Brazil Universidade do Estado do Rio de Janeiro. Rio de Janeiro, Rio de Janeiro, Brazil; II Universidade Federal de Mato Grosso do Sul Três Lagoas Mato Grosso do Sul Brazil Universidade Federal de Mato Grosso do Sul. Três Lagoas, Mato Grosso do Sul, Brazil

**Keywords:** COVID-19, Vulnerable Populations, Social Representations, Nursing, Public Health, COVID-19, Poblaciones Vulnerables, Representaciones Sociales, Enfermería, Salud Pública

## Abstract

**Objectives:**

to analyze the social representations of COVID-19 among residents of a vulnerable community in Rio de Janeiro, to provide insights into healthcare and nursing practices.

**Methods:**

we conducted a mixed-methods study based on the Social Representations Theory (SRT), employing a structural approach. The study was performed with 120 residents from the Rocinha community in Rio de Janeiro. We collected data through a sociodemographic questionnaire and analyzed it using descriptive statistics. Free word associations with the prompt term “COVID-19” were collected and submitted to prototypical and similarity analyses.

**Results:**

The central core of the social representation of COVID-19 was found to comprise the elements “death”, “fear”, “loss”, “disease”, and “suffering”. The similarity graph reinforced the centrality of “death” and “fear” in these representations.

**Final Considerations:**

COVID-19’s social representation in this population centered around negative meanings, highlighting their struggle with the disease and the pandemic’s economic impact on vulnerable groups.

## RESUMEN

**Objetivos:**

analizar las representaciones sociales de una comunidad vulnerable de Río de Janeiro sobre la COVID-19, para proporcionar insumos a las prácticas de atención en salud y enfermería.

**Métodos:**

estudio mixto basado en la Teoría de las Representaciones Sociales, empleando un enfoque estructural. El estudio incluyó a 120 residentes de Rocinha, en Río de Janeiro. Los datos se recopilaron mediante un cuestionario sociodemográfico y se analizaron utilizando estadística descriptiva. También se obtuvieron asociaciones libres de palabras con el término “COVID-19”, sometidas a análisis prototípico y de similitud.

**Resultados:**

el núcleo central de la representación social de la COVID-19 se compone de “muerte”, “miedo”, “pérdida”, “enfermedad” y “sufrimiento”. El gráfico de similitud reforzó la centralidad de “muerte” y “miedo”.

**Consideraciones Finales:**

la representación social de la COVID-19 en esta comunidad vulnerable enfoca significados negativos, destacando su lucha contra la enfermedad y el impacto económico de la pandemia en poblaciones vulnerables.

## INTRODUCTION

The COVID-19 pandemic (Coronavirus Disease-2019)^(1)^ triggered a public health emergency, prompting generalized responses to protect populations. However, there is no such thing as a universal human experience. In Brazil, the term “vulnerability” has been used to highlight the multiple layers that compose it, such as social vulnerability, as certain population segments were more susceptible to the unfolding health crisis. Social vulnerability is related to processes exacerbating exclusion, discrimination, or the weakening of groups or individuals^(2)^.

During the pandemic, COVID-19 was characterized as a highly lethal communicable disease, particularly for those with chronic illnesses. Transmission occurs through contact, droplets, or aerosols. Contact transmission happens through direct contact (e.g., handshakes) with an infected person or contaminated objects and surfaces, followed by touching the eyes, nose, or mouth. Droplet transmission occurs when respiratory droplets expelled by an infected person through coughing or sneezing expose others. Aerosol transmission involves smaller respiratory droplets containing the virus that remain suspended in the air^(3,4)^.

The World Health Organization (WHO) recommended several control strategies to reduce exposure to the virus: COVID-19 vaccination, wearing masks, hand hygiene, ensuring proper ventilation, avoiding crowds, and minimizing close contact, especially in enclosed spaces^(4)^. In Brazil, social distancing measures, such as event cancellations, school closures, quarantine for vulnerable groups (those over 60, pregnant women, and people with chronic illnesses) and the general population, economic shutdowns, and transport restrictions, were widely implemented across most of the country’s states during the first year of the pandemic^(4,5)^.

A significant portion of the global population has suffered the impacts of the COVID-19 pandemic. Biologically, the effects included alterations in blood clotting, cardiopulmonary complications, and a decline in sleep quality. Psychologically, sleep disruptions were influenced by emotional distress caused by anxiety, insomnia, and compulsive eating. Lastly, in the psychosocial realm, the pandemic affected interpersonal relationships, security, financial stability, and individuals’ sense of accomplishment and satisfaction.

The extensive impacts of the COVID-19 pandemic pose a challenge to analyze where inequality has weakened spaces and individuals. The externalization of social inequality, health inequities, and geography revealed the unequal distribution of the virus. The pandemic heightened class, racial, and gender disparities, as well as other vulnerabilities, placing individuals in situations where layers of vulnerability overlap, especially in regions historically governed by patriarchal, oppressive systems rife with inequities^(6)^.

The spread of the coronavirus also reached the favelas of major cities—areas marked by inequality and exclusion with high levels of social vulnerability^(7)^. These communities, like Rocinha, are characterized by irregular water supply, poor waste collection, open sewage, and inadequate sanitation—conditions that lead to insalubrity in residents’ daily lives. Such characteristics are linked to groups undergoing social exclusion. “Vulnerable communities” or “communities in situations of social vulnerability” refer to groups and families living in precarious conditions, as described, with limited means of subsistence and a lack of structured family support. Such a scenario creates a daily reality of social risk, where individuals cannot fully exercise their rights and responsibilities as citizens, lose representation in society, and rely on external aid for survival. In these contexts, access to basic needs is not guaranteed through normal channels of resources and opportunities^(2,7)^.

During the pandemic, social distancing measures were not fully adhered to, as remote work was not feasible for most, and there was widespread job and income loss, overcrowded homes, and small, poorly ventilated living spaces. These factors prevented a significant portion of this population from benefiting from protective actions, increasing the risk of exposure to coronavirus infection^(7)^. Consequently, the essential resources required for implementing preventive measures were not equally available to communities with unfavorable living and working conditions^(8)^.

In July 2020, a study published by the Institute of Applied Economic Research (Ipea) indicated that the majority of COVID-19-related deaths in Rio de Janeiro occurred among residents of the city’s poorest neighborhoods. This finding reinforces the diagnosis of the State’s structural absence and lack of sanitary assistance, further marginalizing communities that were already experiencing significant hardships^(9)^.

The actual social impacts of COVID-19 in favelas went largely unnoticed, as widespread and adequate testing did not occur at the start of the pandemic or for much of its duration. Additionally, many cases went underreported, obscuring the actual magnitude of the pandemic in official statistics. The closest data to this reality came from Fiocruz, informed by local leaders and primary health care (PHC) units^(10)^.

In addition to these issues, which highlight the social determinants of the health-disease process, the pandemic brought personal anguish and abrupt lifestyle changes, resulting in panic, fear, doubts, and uncertainties. People saw their freedom restricted, trust eroded, health systems’ capacity jeopardized, and the economy weakened^(11)^.

These conditions could have physical and mental health consequences, leading to lifestyle changes such as reduced physical activity, increased consumption of alcohol, cigarettes, drugs, and unhealthy foods, as well as heightened stress and anxiety^(12)^.

Given the psychosocial impacts of the pandemic, we chose to investigate the perceptions of a social group regarding COVID-19 based on Social Representations Theory (SRT). Social representations are considered a specific type of social thought, consisting of dynamic sets to interpret reality. They guide communication, understanding, and control of the environment—not only social but also material and ideal. SRT is primarily concerned with studying social symbols and how these symbols influence the construction of shared knowledge and culture^(13)^.

By defining SRT as the guiding framework for this study, we adopt the postulate that all social reality is represented and appropriated individually or collectively, reconstituted by the cognitive system, and integrated into each subject’s value system, which depends on their history and the social and ideological context in which they are embedded^(14)^.

## OBJECTIVES

To analyze the social representation of COVID-19 among residents of a vulnerable community in Rio de Janeiro, with the goal of providing insights into healthcare and nursing practices.

## METHODS

### Ethical aspects

The Research Ethics Committee of UERJ approved this project. In adherence to research ethics, we ensured participants’ rights to confidentiality, anonymity, and the option to withdraw at any point. We also made clear the absence of any costs or benefits for participants. The possible risks of this research were limited to psychological discomforts that could arise during data collection. After agreeing to participate in the study, each participant was given with two copies of the Informed Consent Form (ICF) for signature, one for themselves and one for the researcher.

### Study type and theoretical-methodological framework

This descriptive study employs a mixed-methods approach, utilizing two data analysis techniques related to descriptive statistics. Additionally, inferential qualitative analysis was performed to interpret the meanings of the evoked words and their significance within the representational structure. We based this study on SRT^(14)^, utilizing the structural approach, which posits that social representation consists of a central system and a peripheral system, complemented by contrasting elements^(15)^.

This study is part of the research project “The Social Construction of Coronavirus and COVID-19 and Its Lessons for Personal, Professional, and Social Care Practices”, funded by CNPq Proc. 422312/2021-5 and FAPERJ Proc. E-26/211.849/2021.

### Study setting

We conducted the study in the Rocinha community in the São Conrado neighborhood in Rio de Janeiro (RJ). Rocinha is characterized by a high population density, precarious housing conditions, irregular water supply, inadequate waste collection, open sewage systems, and insufficient public services. The community is also marked by daily experiences of violence, inequality, and exclusion, classifying it as a highly socially vulnerable community. During the pandemic, social distancing measures were not fully adhered to, as remote work was not possible for most residents, resulting in job and income loss, overcrowded living conditions, and limited ventilation. These factors prevented a significant portion of the population from benefiting from protective measures, increasing the risk of coronavirus exposure. Consequently, the basic resources necessary for preventing coronavirus contagion were not equally available to this community.

### Data source

The study participants were 120 residents of the Rocinha community, aged 18 or older, who were autonomous in their participation during data collection. We intentionally selected participants for the study, involving the first individuals who agreed to participate on the scheduled data collection days. Invitations were extended to Primary Health Care service users in the participating community.

### Data collection, organization, and analysis

We collected sociodemographic and clinical data using a form that included variables related to the participants’ social characteristics and the occurrence of COVID-19. Additionally, we employed the free word association technique, with “COVID-19” as the stimulus word, without requiring participants to justify their word choices. This decision meant that the researchers, following a jury dynamic, interpreted the meanings expressed by the words. This method allowed three participating researchers to agree on the interpretation of the words present in the quadrants to establish the representational structure. Research team meetings were held to discuss the constructed quadrants. In cases of disagreement over the meaning of specific words, the final interpretation was determined based on the agreement of two out of the three researchers.

Data collection took place from July to November 2021, 15 months after the onset of the COVID-19 pandemic in Brazil, which recorded its first confirmed case on February 26, 2020, when vaccination had already begun. The collection was conducted individually, with the interviewer filling out a form based on the verbalization of five words or expressions that came to the interviewee’s mind after the interviewer stated the stimulus term.

We analyzed the sociodemographic and clinical data using descriptive statistics through the Statistical Package for the Social Sciences (SPSS) software, which provided the simple and relative frequencies of the variables. For the data collected through the free word association technique, we used the EVOC software (*Ensemble de Programmes Permettant L’Analyse des Évocations*)^(16)^, which enables the construction of the Four Quadrant Chart. This chart is built using two indicators of importance: the frequency of appearance and the order in which each word was evoked. These are expressed, respectively, by the average frequency of the set of words and the weighted average of the order in which each word was evoked. The chart synthesizes the structure and representational content, distributing them into four quadrants. The combination of these criteria defines the boundaries of each quadrant, allowing us to identify words that are most likely to belong to the central core due to their prototypical nature, as well as peripheral and contrasting elements^(15)^.

Words with higher frequencies and those evoked more readily, located in the upper-left quadrant, are considered the probable elements of the central core of the social representation. These elements are stable, do not vary based on the immediate context, and provide the primary meaning to the representation, where they serve organizing and stabilizing functions. Not all elements in this quadrant are central, but the central core of the representation is found there, requiring the association of techniques for confirmation. Words with higher or lower frequencies, evoked less readily and found in the upperright and lower-right quadrants, make up the peripheral system. These elements serve to shape and regulate the central core, supporting the heterogeneity of the group’s positions and acting to prevent changes in the central core while establishing connections with social practices. The fourth quadrant, in the lower left, contains the contrast zone, with elements that are evoked readily but have lower frequencies than the average. This zone may reveal the existence of subgroups with a distinct central core from the general group or simply elements that indicate dimensions already revealed in other quadrants, thus complementing the others^(15)^.

To strengthen the structural analysis and confirm the centrality of the elements, we used a second technique: similarity analysis. This method assesses the number of connections an element has with other evoked elements, expressing these links through similarity indices, resulting in a similarity graph. This graph visually represents the connections between the elements evoked by the group^(17,18)^. This approach aimed to understand the associations between the elements that comprise the social representation of COVID-19 as a second indication of the centrality of elements identified by the prototypical analysis.

## RESULTS

Among the 120 participants, 74 (61.67%) were female, aged 18 to 76. The most represented age group was between 30 and 39 (38.33%). Regarding education, most participants had completed high school (43; 35.8%). In terms of spiritual beliefs, the majority of participants (49; 40.8%) are Catholic. The predominant work field was industry and commerce (50; 41.7%). The income of 71 participants (59.2%) ranged from R$ 1,001.00 to R$ 2,000.00, which represented 0.91 to 1.8 minimum wages at the time (R$ 1,100.00). The majority of participants reported having no political affiliation (101; 84.2%), and 63 (52.5%) indicated that they had contracted COVID-19.

In the prototypical analysis of free word associations, we identified 596 cited words, with 117 unique words. For content processing, we set the minimum frequency at seven words, in line with Zipf’s Law(16), excluding terms evoked less frequently. We calculated the average frequency of evocation (AFE) as 18, and the average order of evocation (AOE) was 3.00.

We describe and analyze the results of the prototypical analysis based on the theoretical assumptions of SRT in its structural approach (Chart 1).

**Chart 1:** Four Quadrant Chart of evocations to the stimulus term “COVID-19” for residents of a vulnerable community in Rio de Janeiro, Rio de Janeiro, Brazil, 2022 (N = 120)

AOE < 3.00				AOE ≥ 3.00		
AFE	Evoked term	F	AOE	Evoked term	f	AOE
≥ 18	***death*** ***fear*** loss **illness** suffering	70 57 21 18 18	1.900 2.456 3.000 2.536 2.833	sadness	37	3.297
< 18	despair pain ***isolation*** **prevention** *hunger* ***hospital*** **vaccine** *poverty*	17 11 11 10 9 9 9 7	2.941 2.909 3.000 2.900 2.333 2.778 2.667 2.571	anxiety **cure** ***mask*** *unemployment* dread longing **care** worry	15 15 12 11 8 8 7 7	3.467 4.000 3.333 3.182 4.125 4.125 3.143 4.429

***Informational or conceptual dimension*** *; **representational field or image dimension**; social dimension; affective-attitudinal dimension.*

In line with Chart 1, the upper left quadrant, representing the central core, reveals a negative affective-attitudinal orientation toward COVID-19, reflected in the terms “fear,” “loss”, and “suffering”. In addition, a conceptual dimension related to incorporated information about COVID19 is expressed in the terms “death” and “illness”. The most frequently cited term was “death”, with a frequency of 70, the highest across all quadrants in the Four Quadrant Chart. However, it had the lowest AOE, indicating it was the most readily evoked element. The second most frequently evoked term was “fear”, with a frequency of 57, while “loss” had the third highest frequency at 21. The elements “illness” and “suffering” were cited less frequently in the probable central core of the social representation.

In the upper and lower right quadrants, which constitute the peripheral system, the word “sadness” appeared 37 times, making it the only component in the first periphery. The second periphery included “anxiety”, “cure”, “mask”, “unemployment”, “dread”, “longing”, “care”, and “worry”. Notable elements of the peripheral system were “anxiety” and “cure”, both of which had high frequencies^(15)^ within the quadrant. Except for the terms “cure”, “care”, and “mask”, the other words carried a negative connotation, reinforcing the overall negative attitude toward COVID-19. The terms “care” and “worry” had the lowest frequencies in the quadrant.

In the lower left quadrant, representing the contrast zone, we find “despair”, “pain”, “isolation”, “hunger”, “hospital”, “vaccine”, and “poverty”, which reflect dimensions already present in other quadrants. These words serve as a complement to the central core, reinforcing its meanings and pointing to an alternative representation. However, the social dimension stands out more in this quadrant than others, as expressed through the words “hunger” and “poverty”.

**Figure 1:**
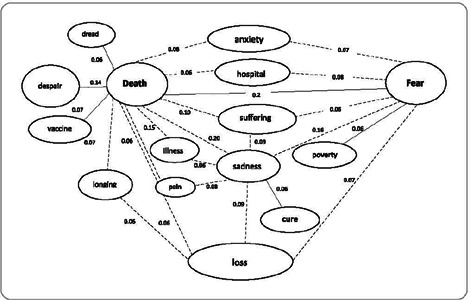
Similarity graph of free word associations with the term “COVID-19”, Municipality of Rio de Janeiro, Rio de Janeiro, Brazil, 2022 (n = 115)

We observe a set of words and their meanings that were evoked with higher frequencies and more readily (lower orders of citation), consisting of “death” and “fear ”. These are accompanied by the meaning expressed by “suffering” and “sadness”, which together represent the third highest frequency in the quadrant. This suggests that the social representation of COVID-19 for Rocinha residents revolves around the meaning of the “death-fear-suffering” triad (Figure 1).

The similarity analysis allows for graphic visualization of the strongest connections established between the elements in the Four Quadrant Chart. It corroborates the centrality of the terms in the social representation of COVID-19 and expresses the most interconnected representational contents (Figure 1).

The similarity graph shows three main sense cores (or nodes), characterized by words with the most connections. These cores are expressed by the terms “death”, with 12 connections; “fear”, with 7 connections; and “sadness”, with 7 connections. The word “death” acts as an organizing term for the other elements, both through the number of connections and the strength of those connections to associated terms: “fear (0.29)”, “sadness (0.20)”, “illness (0.15)”, “loss (0.15)”, “despair (0.14)”, “suffering (0.10)”, “anxiety (0.08)”, “vaccine (0.07)”, “longing (0.07)”, “dread (0.06)”, “hospital (0.06)”, and “pain (0.06)”. The term “fear” is the second most connected element (seven associated elements) and is linked with “death” (0.29), “sadness” (0.16), “hospital” (0.08), “loss” (0.07), “anxiety” (0.07), “suffering” (0.06), and “poverty” (0.06). These two elements were central in the prototypical analysis (Figure 1) and confirmed their centrality in the similarity analysis.

The term “sadness” had seven connections, with similar connection values to “death” (0.20), “fear” (0.16), “suffering” (0.09), “loss” (0.09), “pain” (0.08), “illness” (0.06), and “cure” (0.06). This configuration suggests that “sadness” could be a central element despite its location in the first periphery of the Four Quadrant Chart. It also supports the earlier proposition that “sadness” expresses the same meaning as “suffering” (central in the prototypical analysis). The other elements involved in the similarity analysis confirm their positions in the prototypical analysis.

## DISCUSSION

The results indicate a social reality shaped and understood through the sociocognitive system of participants from vulnerable communities in Rio de Janeiro, based on their values, experiences, and social and cultural context.

Moscovici^(19)^ proposes that the organization of a social representation occurs in three dimensions: the informational or conceptual dimension, the representational field or image, and the affective-attitudinal or judgment-related dimension. These dimensions are described as follows:

Information refers to the knowledge a group has about a social object, varying in terms of quantity and quality. The representational field points to the idea of an image or social model, encompassing the concrete and limited propositions regarding a specific aspect of the represented object. It is linked to the structured elements of the representation. Attitude, the most common dimension, involves the behavioral orientation toward the social representation object. It guides and influences the behaviors related to the object and provokes emotional reactions, engaging people with varying degrees of intensity^(19)^.

Based on these assumptions, the analysis of Chart 1 reveals that the possible central core of the social representation consists of meanings associating COVID-19 with death, fear, illness, loss, and suffering. These symbolic contents reflect the participants’ experience and awareness of the disease’s severity, highlighting negative feelings such as insecurity and anguish due to its high mortality rate. Thus, the central core is characterized by normative elements, namely death and fear. These elements were activated based on the group’s assessment or judgment about COVID-19, guiding the psychosocial construction of the disease. Furthermore, the core comprises functional elements (loss, illness, and suffering) associated with lived experiences during the pandemic.

The connection between the core terms suggests that COVID-19 is perceived as a disease leading to death, which functions both as a conceptual and imagistic dimension, evoking feelings of suffering, loss, and fear—constituting a negative affective-attitudinal dimension.

Regarding the affective dimension, which some authors call the “attributive dimension,” emotions are “the emotive coloring that permeates human existence and, in particular, the relationship with the world”^(20)^. It encompasses the emotions and feelings present in judgments or behaviors triggered by the evaluation of the social object. In this study, the affective dimension is predominantly negative, reflecting the panic induced by an unknown, lethal, and highly transmissible disease.

These elements are grounded in the high mortality rate COVID-19 presented in Brazil through the first half of 2022. Death is a distressing and frightening situation, and the pandemic’s rapid and frequent fatalities had a significant impact on the psychosocial constructions of the studied group—constructions expressed through negative emotional content. The term “death” appeared most frequently, was the most readily evoked, and had the most connections with other elements, indicating its structuring role in the representation of COVID-19. It was associated with affective terms such as fear, sadness, loss, despair, suffering, anxiety, longing, dread, and pain. It is only tangentially connected to terms linked to a clinical-biomedical perception of the pandemic, such as illness, hospital, and vaccine, revealing death’s strong association with psychological and emotional components.

Fear also played a central role, forming significant connections with “death” and other psychological and emotional elements like “sadness”, “loss”, “anxiety”, and “suffering”, as well as with “hospital” and “poverty”. This reinforces fear as a structuring element of the representation. Fear may be tied to the real and immediate possibility of coronavirus infection, leading to illness and possibly death, thus highlighting the imminent and symbolic threat of the virus. The fear dimension also reflects experiences associated with gaps in knowledge about COVID-19, including its transmission, pathophysiology, and people’s vulnerability, which led to significant risks, fear, and apprehension^(21)^.

The cognemes “loss” and “suffering” reflect psycho-emotional dimensions connected to death, relating to the end of life or the loss of relationships with friends, family, and loved ones.

Central elements serve to organize and stabilize a social object’s representation, giving it meaning. The content of the central core consists of stable elements that do not vary with the immediate context. This core is determined by the nature of the represented object, the type of relationship the group maintains with it, and the system of social values and norms shaping the group’s ideological environment^(15)^.

In the peripheral system, the term “sadness” forms strong connections with “death” and “fear”, expressing the affective-attitudinal dimension of the social representation of COVID-19. This negative term was activated by the social object being analyzed^(22)^. It relates to the lived experiences expressed in the core elements of “death”, “loss”, and “suffering”. It may also relate to the metaphor of social death, as COVID-19 forced individuals to withdraw from their social support networks and altered their societal roles. This representational dimension reflects the emotional experiences the participants faced, generating stress and suffering^(23)^.

In the peripheral system, the second periphery of the social representation of COVID-19 includes not only normative elements but also functional ones. Additionally, it expresses conceptual and affective dimensions and introduces a new social dimension in the peripheral system, reflected in the term “unemployment”. The elements in the second periphery suggest an articulation with the probable central system, especially the term “longing”, which is associated with the ideas of death and loss, as well as anxiety, dread, and worry, all connected to the fear and suffering induced by COVID-19. These terms seem consistent with “sadness,” located in the first periphery of the representation. Together, they function as protective elements for the central core, emphasizing the predominance of negative feelings.

The peripheral elements play a crucial role in shaping and regulating the central core, supporting the heterogeneity of positions expressed by the group. Thus, we observe coherence between the central core and the periphery in this analysis^(15)^.

The terms “cure”, “mask”, and “care” correspond to the central element “illness”. These terms represent a conceptual dimension linked to the reified universe of the coronavirus and COVID-19. Highlighting the functional elements of the representation, these cognemes reflect the group’s actions to combat the disease and death, serving as strategies to prevent infection. Similar findings were reported in a study conducted in the Northern region of Brazil, where alcohol, soap, masks, and isolation were identified as protective measures against the spread of the virus^(23)^.

The term “unemployment” does not correspond to the central core or first periphery, pointing to a new dimension of social impacts caused by the pandemic within this representation. This dimension becomes more evident in the contrast zone of the prototypical analysis, particularly through the elements of hunger and poverty. These terms illustrate how COVID-19 exacerbated social inequalities, increased unemployment, reduced wages, and deepened social exclusion. They highlight the economic impact of the disease on society, especially among vulnerable social classes, exposing the cruel reality that extended beyond the physical and psychological symptoms of the virus. Additionally, they reveal the group’s pre-existing vulnerability due to the social inequality present even before the pandemic.

Research indicates that the pandemic’s impact was not limited to public health but also led to increased unemployment and significantly affected the labor market, particularly among young people^(24,25)^. Families already living in vulnerable conditions became more susceptible due to accelerated unemployment, precarious or informal work, and declining income, along with unsanitary living conditions. As a result, they became more reliant on short-term social programs for support^(26)^. Beyond the pragmatic effects, unemployment disrupts identity and social belonging, with consequences for mental health and social identity during a time of extreme fragility^(27)^.

The remaining elements in the contrast zone reaffirm the perceptions presented in the other quadrants. They include normative and functional elements, with the affective dimension represented by the words “despair” and “pain”, connected to the core elements of “death”, “loss”, and “suffering” as well as to “sadness” in the first periphery and “dread”, “longing”, and “anxiety” in the second periphery. These elements inevitably convey a representation dominated by negative emotions.

The terms “isolation”, “prevention”, “hospital”, and “vaccine” relate to the elements “cure”, “mask”, and “care”, reflecting measures to prevent and confront the disease and potential death. These elements illustrate the reified universe of scientific knowledge. They reveal the participants’ familiarity with necessary measures to prevent infection and its worsening, highlighting the conceptual (informative) dimension as well as the functional elements of the COVID19 representation.

The term “vaccine” was connected to the lexicon “death” in the similarity graph, representing a countermeasure against mortality and a protective strategy to prevent illness and death. The reference to hospitals as associated with death and fear reflects how this symbolic construction was shaped largely by media images portraying healthcare units as war zones or death fields.

The results show that the group’s psychosocial construction of COVID-19 was based on the perception of it as a destructive and lethal disease that causes loss and suffering. The structuring elements of this representation are “death”, “fear”, and “suffering”, forming its central core. However, despite the negative central core, neutral elements related to disease management, preventive measures, and practices to avoid infection also emerged, reflecting resilience in a destructive scenario.

COVID-19 presented significant challenges for public health, thereby overwhelming hospitals and health institutions and affecting various workforces^(28)^. Amid efforts to combat the pandemic, some activities lost prominence in nurses’ daily routines, including health education, which is typically a participatory and emancipatory practice aimed at raising awareness and promoting collective and individual action to improve quality of life^(29)^.

The workload resulting from the immediate impact of COVID-19 on healthcare services reduced educational activities, creating gaps that allowed misinformation and fake news to spread on social media. This fact changed symbolic elements already established in social groups, such as adherence to immunization under the National Vaccination Program.

Nurses faced the challenge of creatively delivering health education, as it is the primary means by which reliable information is conveyed to support social practices of self-protection and the protection of others^(29)^. Health education remains a fundamental care activity, encouraging reflection and breaking paradigms, potentially provoking changes in representations and practices related to COVID-19.

The representational elements presented in this study reveal the emotional suffering experienced by those who lived through the pandemic. This event generated grim outlooks, social abandonment, and emotional distress, leaving the most vulnerable at higher risk of developing psychological symptoms, potentially leading to psychopathological conditions. In such circumstances, health education becomes increasingly important. It is intended for the general population and must be considered an essential activity in planning pandemic responses, as it influences lifestyle and behaviors developed in response to the lived reality^(30)^.

In this context, considering that PHC is a setting for providing effective practices and care, nursing practices should focus on addressing the biopsychosocial needs of individuals beyond just physical health and the biological body. Care strategies like welcoming, attentive listening, and comforting patients are complex approaches that underpin health care. Similarly, matrix support, home visits, therapeutic groups, and telehealth services enhance the relationship with care and health management, potentially reducing suffering in pandemic situations.

The social representation of COVID-19 exposed the impact of social inequality on health, economic vulnerability, and poverty experienced by the group, leading to greater social ruptures than those already present before the pandemic. The individual and collective measures imposed by the pandemic, particularly for the most vulnerable groups, compromised the basic provisions necessary for daily survival, quality of life, and disease prevention, leading to even more precarious health and living conditions. This scenario placed constraints on comprehensive care and the healthcare system, which struggled to find solutions to mitigate the pandemic’s effects. The sector relies on the robust presence of the State in implementing and enhancing income transfer policies and strengthening public health through the Unified Health System (SUS).

### Study limitations

The primary limitation of this study was the sample size, which prevented a comparative analysis between individuals who reported contracting COVID-19 and those who did not. Such a comparison could have enriched the discussion by highlighting variations in the social construction of the disease based on greater or lesser personal contact with it.

### Contributions to the field of nursing

This study provided valuable insights into the psychosocial realities experienced during the COVID-19 pandemic. It helped to understand the experiences of social actors from socially vulnerable groups and offered support for developing healthcare practices in a pandemic context.

## FINAL CONSIDERATIONS

The study revealed that the social representation of COVID-19 is characterized by death, fear, and sadness as its core elements. This representation’s internal structure reflects coherence between central and peripheral elements, shedding light on how the group negatively perceived COVID-19 as a deadly disease associated with fear, suffering, sadness, and longing. Furthermore, the research showed functional elements representing the group’s practices for combating the disease, such as mask usage, isolation, and vaccination. It also highlighted poverty and hunger as social and economic outcomes of the pandemic, predominantly affecting vulnerable groups like residents of favelas.

This analysis of the social representation of COVID-19 underscores the need to improve healthcare services provided by nurses and other healthcare professionals. It demonstrated that reconfiguring care for this population is essential, considering not only the physical effects of the disease but also its psychosocial impacts.

## References

[1] Organização Pan-Americana da Saúde (OPAS). Atualização epidemiológica: COVID-19 doença causada pelo novo coronavírus [Internet]. Washington, DC: OPAS; 2020[cited 2023 Oct 3]. Available from: https://www.paho.org/pt/documentos/atualizacao-epidemiologica-covid-19-doenca-causada-pelo-novo-coronavirus-18-setembro

[2] Ximenes DA. Vulnerabilidade social. In: Oliveira DA, Duarte AMC, Vieira LMF. Dicionário: trabalho, profissão e condição docente. Belo Horizonte: UFMG, Faculdade de Educação; 2010.

[3] Ministério da Saúde (BR). Orientações para manejo de pacientes com COVID-19 [Internet]. 2020[cited 2023 Oct 3]. Available from: https://www.gov.br/saude/pt-br/assuntos/covid-19/publicacoes-tecnicas/recomendacoes/orientacoes-para-manejo-de-pacientes-com-covid-19/@@download/file

[4] Organização Mundial da Saúde (WHO). WHO Guideline on Self-Care Interventions for Health and Well-Being: 2022 revision [Internet]. Geneva: WHO; 2022[cited 2023 Oct 3]. Available from: https://www.who.int/publications/i/item/9789240052192

[5] Silva MAS, Lima MCL, Dourado CARO, Pinho CM, Andrade MS. Biossegurança dos profissionais de enfermagem no enfrentamento da COVID-19. Rev Bras Enferm. 2022;75(1):1-7. https://doi.org/10.1590/0034-7167-2020-1104

[6] Oliveira RG. Práticas de saúde em contextos de vulnerabilização e negligência de doenças, sujeitos e territórios: potencialidades e contradições na atenção à saúde de pessoas em situação de rua. Saúde Soc. 2018 ;27(1):37-50. https://doi.org/10.1590/S0104-12902018170915

[7] Lima ALS, Périsse ARS, Leandro B, et al. Covid-19 nas favelas: cartografia das desigualdades. In: Matta GC, Rego S, Souto EP, et al, Os impactos sociais da Covid-19 no Brasil: populações vulnerabilizadas e respostas à pandemia. Rio de Janeiro: Observatório Covid 19 Editora Fiocruz. 2021;25(7):111-2. https://doi.org/10.7476/9786557080320.0009

[8] Khalidi JR. Inequality Affects the COVID-19 Pandemic. KRI Views [Internet]. 2022 [cited 2023 Oct 3];22(20):15-19. Available from: http://www.krinstitute.org/assets/contentMS/img/template/editor/20200330_Articles_Covid_Inequality_v9.pdf

[9] Evangelista AP. COVID-19 Favelas: Fiocruz aponta que pandemia tem mais impacto em áreas pobres[Internet]. Escola Politécnica de Saúde Joaquim Venâncio; 2021 [cited 2023 Oct 3]. Available from: https://www.epsjv.fiocruz.br/podcast/covid-19-favelas-fiocruz-aponta-que-pandemia-tem-mais-impacto-em-areas-pobres-do-rio

[10] Fundação Oswaldo Cruz. Observatorio Covid. Impacto Social da COVID-19. Favelas na luta contra o coronavírus[Internet]. 2021 [cited 2023 Oct 3]. Available from: https://www.educare.fiocruz.br/resource/show?id=a2U63Tpj

[11] Aquino EML, Silveira IH, Pescarini JM, Aquino R, Souza-Filho JA, Rocha AS, et al. Medidas de distanciamento social no controle da pandemia de COVID-19: potenciais impactos e desafios no Brasil. Ciênc Saúde Coletiva. 2020;25(suppl):2423-46. https://doi.org/10.1590/1413-81232020256.1.10502020 10.1590/1413-81232020256.1.10502020 32520287

[12] Malta DC, Gomes CS, Szwarcwald CL, Barros MBA, Silva AG, Prates EJS, et al. Distanciamento social, sentimento de tristeza e estilos de vida da população brasileira durante a pandemia de COVID-19. Saúde Debate. 2020;44(esp. 4):177-90. https://doi.org/10.1590/0103-11042020E411

[13] Jodelet D. Folies et représentations sociales. 3. ed. Paris: PUF; 2005.

[14] Vergès P. Ensemble de programmes permettant l’analyse des evocations (EVOC 2005): manuel version 6/2006. Aix-en-Provence: LAMES, 2006.

[15] Abric JC. Méthodes d’études des représentations sociales. Saint Agne: Erè; 2003.

[16] Moscovici S. Representações sociais: investigações em psicologia social. Petrópolis: Vozes; 2013.

[17] Pecora AR, Sá CP. Memórias e representações sociais da cidade de Cuiabá, ao longo de três gerações. Psicol: Reflex Crít. 2008;21(2):319-25. https://doi.org/10.1590/S0102-79722008000200018

[18] Pontes APM, Oliveira DC, Gomes AMT. Os princípios do Sistema Único de Saúde estudados a partir da análise de similitude. Rev Latino-Am Enfermagem. 2014;22(1):59-67. https://doi.org/10.1590/0104-1169.2925.2395 10.1590/0104-1169.2925.2395 PMC4292688 24553704

[19] Moscovici S. Social cognition: perspectives on everyday understanding. London: Academic Press; 1978.

[20] Arruda A. Meandros da teoria: a dimensão afetiva das representações sociais. In: Almeida AMO, Jodelet D. (org.) Interdisciplinaridade e diversidade de paradigmas. Brasília: Thesaurus; 2009. p.83 – 102.

[21] Almeida RMF, Antunes LMS, Barros FM, Silva RC. Covid-19: um novo fenômeno de representações sociais para a equipe de enfermagem na terapia intensiva. Esc Anna Nery. 2021 ;25(spe):e20200118. https://doi.org/10.1590/2177-9465-EAN-2020-0118

[22] Campos PHF, Rouquette ML. Abordagem estrutural e componente afetivo das representações sociais. Psicol: Reflex Crít. 2003;16(3):435-5. https://doi.org/10.1590/S0102-79722003000300003

[23] Do Bú EA, Alexandre MES, Bezerra VAS, Sá-Serafin RCN, Coutinho MPL. Representações e ancoragens sociais do novo coronavírus e do tratamento da COVID-19 por brasileiros. Estud Psicol. 2020;37(e200073). https://doi.org/10.1590/1982-0275202037e200073

[24] Silva CP, Albuquerque FDN, Lopes BJ. Social representations of unemployment, mental health and the covid-19 pandemic in a small Brazilian sample. Braz J Health Rev. 2021;4(2):7249-62. https://doi.org/10.34119/bjhrv4n2-269

[25] Costa SS. Pandemia e desemprego no Brasil. Rev Adm Pública. 2020;54(4):969-78. https://doi.org/10.1590/0034-761220200170

[26] Ribeiro-Silva RC, Pereira M, Campello T, Ferreira AJF, Santos SMC, Aragão E, et al. Implicações da pandemia COVID-19 para a segurança alimentar e nutricional no Brasil. Ciênc Saúde Coletiva. 2020;25(9):3421-30. https://doi.org/10.1590/1413-81232020259.22152020 10.1590/1413-81232020259.22152020 32876253

[27] Schmidt MLG, Januário CARM, Rotoli LUM. Sofrimento psíquico e social na situação de desemprego. Cad Psicol Soc Trab. 2018;21(1):73-85. https://doi.org/10.11606/issn.1981-0490.v21i1p73-85

[28] Machado MH, Machado AV, Teixeira EG, Militão JB, Barbosa S, Leonel F. A pandemia prolongada e os trabalhadores da saúde no front: uma encruzilhada perigosa. Centro de Estudos Estratégicos da Fiocruz [Internet]. 2022[cited 2022 Jan 3] Available from: https://informe.ensp.fiocruz.br/noticias/52640

[29] Ministério da Saúde (BR). Temática promoção da saúde IV. Brasília (DF): Organização Pan-Americana da Saúde; 2009.

[30] Barbosa NS, Costa APC, Ribeiro AA, Rocha EP, Ribeiro PVS, Fernandes MA. Práticas de autocuidado em saúde mental de enfermeiros na pandemia da Covid-19. Rev Enferm Atual Derme. 2023;97(2):e023116. https://doi.org/10.31011/reaid-2023-v.97-n.2-art.1717

