# Supplementary material for: The symbolic-representational construction of COVID-19 in vulnerable population groups
Source: Rev Bras Enferm. 2025 Mar 17;78(Suppl 2):e20240120. doi: 10.1590/0034-7167-2024-0120 (PMC11913423; doi:10.1590/0034-7167-2024-0120)
Supplement: Translation

# Translation: A construção simbólico-representacional da COVID-19 em grupos populacionais vulneráveis

**DOI:** 10.1590/0034-7167-2024-0120pt

**Authors:** Denize Cristina de Oliveira, Vanessa Bittencourt Ribeiro, Yndira Ita Machado, Sergio Correa Marques, Jessica Grativol Aguiar Dias de Oliveira, Hellen Polliana Cecílio, Renata Lacerda Marques Stefaisk, Juliana Pereira Domingues

**Affiliations:** I Universidade do Estado do Rio de Janeiro. Rio de Janeiro, Rio de Janeiro, Brasil; II Universidade Federal de Mato Grosso do Sul. Três Lagoas, Mato Grosso do Sul, Brasil

**Keywords:** COVID-19, Populações Vulneráveis, Representações Sociais, Enfermagem, Saúde Pública

## Abstract

**Objetivos:**

analisar as representações sociais dos moradores de uma comunidade vulnerável do Rio de Janeiro sobre a COVID-19, com vistas a fornecer subsídios para as práticas de cuidado de saúde e de enfermagem.

**Métodos:**

estudo qualiquantitativo, fundamentando-se na Teoria das Representações Sociais e utilizando a abordagem estrutural. Foi realizado com 120 moradores da comunidade da Rocinha – Rio de Janeiro. Os dados foram coletados mediante um questionário sociodemográfico e analisados com estatística descritiva. Foram coletadas evocações livres ao termo indutor “COVID-19”, submetidas às análises prototípica e de similitude.

**Resultados:**

o núcleo central da representação social da COVID-19 é constituído pelos elementos “morte”, “medo”, “perda”, “doença” e “sofrimento”. O grafo de similitude reforçou a centralidade da morte e do medo.

**Considerações Finais:**

a representação foi construída em torno de significados negativos e de elementos que apontam o enfrentamento da doença e o impacto econômico da COVID-19 nas comunidades vulneráveis.

## INTRODUÇÃO

A pandemia de COVID-19 (Coronavirus Disease-2019)^(1)^ produziu uma emergência em saúde pública, acarretando respostas para a proteção das populações de forma genérica; no entanto, não há um humano universal. No Brasil, o termo “vulnerabilidade” foi utilizado expondo as diversas camadas que a compõe, como a vulnerabilidade social, já que determinados segmentos populacionais foram mais suscetíveis à crise sanitária instalada. A vulnerabilidade social é um conceito relacionado aos diversos processos que acentuam a exclusão, a discriminação ou o enfraquecimento de grupos ou indivíduos^(2)^.

Durante a pandemia, a COVID-19 caracterizou-se como doença transmissível altamente letal, especialmente entre pessoas acometidas por doenças crônicas, cuja transmissão pode ocorrer por meio de contato, gotícula ou aerossol. A transmissão por contato acontece mediante o contato direto (p.ex., aperto de mão) com uma pessoa infectada ou com objetos e superfícies contaminados, seguido do toque nos olhos, nariz ou boca. A transmissão por gotículas ocorre pela exposição a gotículas respiratórias expelidas por uma pessoa infectada por meio da tosse ou espirro. Já a transmissão por aerossol acontece por meio de gotículas respiratórias menores que contêm o vírus e permanecem suspensas no ar^(3,4)^.

As estratégias de controle ou redução de exposição ao vírus recomendadas pela OMS foram: vacinar contra a COVID-19, usar máscaras, higienizar as mãos, manter os ambientes ventilados, evitar aglomerações e reduzir o contato próximo com outras pessoas, principalmente em espaços fechados^(4)^. No Brasil, as medidas de distanciamento social, abrangendo suspensão de eventos e aulas, quarentena para grupos vulneráveis (acima de 60 anos, gestantes e pessoas com doenças crônicas) e população em geral, paralisação econômica e restrição de transporte, foram largamente implementadas na grande maioria das Unidades da Federação (UF), no primeiro ano de pandemia^(4,5)^.

Grande parte da população mundial tem sofrido os impactos da pandemia de COVID-19. Na esfera biológica, os efeitos incluíram alterações na coagulação sanguínea, acometimento cardiorrespiratório e piora na qualidade do sono. Já na esfera psíquica, o sono prejudicado foi influenciado pelo sofrimento psíquico decorrente de ansiedade, insônia e compulsão alimentar. Por fim, os problemas na esfera psicossocial englobaram as relações interpessoais, de segurança e de estabilidade financeira e o nível de realização e satisfação dos indivíduos.

A amplitude dos impactos da pandemia de COVID-19 impõe o desafio de analisar o local onde o processo de desigualdade enfraqueceu espaços e sujeitos. A exteriorização da desigualdade social, das iniquidades em saúde e da geografia expôs a distribuição desigual do vírus. A doença acentuou diferenças de classes, raça, gênero e outras fragilidades da vida humana, colocando as pessoas em situações nas quais camadas de vulnerabilidade vão sobrepondo-se, especialmente em locais historicamente governados por sistemas patriarcais, opressores e repletos de iniquidades^(6)^.

A propagação do coronavírus atingiu, também, as áreas de favela nas grandes cidades, que são espaços marcados pela desigualdade e pela exclusão com alta vulnerabilidade social^(7)^. Esses cenários de comunidades e favelas, como a da Rocinha, são caracterizados pelo fornecimento irregular de água, coleta de lixo deficiente, esgoto a céu aberto e más condições de saneamento básico; enfim, condições que provocam insalubridade no cotidiano dessas populações. Tais características se associam a grupos em processo de exclusão social. Entende-se por “comunidades vulneráveis ou em situação de vulnerabilidade social” aquelas que agregam pessoas e famílias vivendo em condições de vida precárias, conforme apontado, associadas a meios de subsistência limitados e à falta de suporte familiar estruturado. Tal cenário aponta um cotidiano de risco social em que o indivíduo não consegue exercer plenamente seus direitos e deveres como cidadão, perde a representatividade na sociedade e depende de auxílios externos para sobreviver. Nesse contexto, a garantia de atendimento das necessidades básicas não é assegurada pelas vias normais de acesso a recursos e oportunidades^(2,7)^.

Durante a pandemia, medidas de distanciamento social não foram cumpridas, pois, na comunidade de modo geral, houve impossibilidade de exercer trabalho remoto, perda de emprego e de fontes de renda, aglomerações nas próprias casas, além de o espaço territorial ser pequeno e pouco arejado. Essa situação impediu que parte significativa desse contingente populacional se beneficiasse de ações protetivas, o que aumentou o risco de exposição ao contágio pelo coronavírus^(7)^. Portanto, os recursos básicos necessários às medidas de prevenção do contágio não estavam igualitariamente disponíveis para comunidades com condições de vida e trabalho desfavoráveis^(8)^.

Em julho de 2020, um levantamento divulgado pelo Instituto de Pesquisa Econômica Aplicada (Ipea) indicou que a maioria das mortes causadas pela COVID-19 no Rio de Janeiro foi observada entre moradores dos bairros mais pobres. Isso reforça o diagnóstico de ausência estrutural do Estado e falta de assistência sanitária vulnerabilizando ainda mais comunidades que já apresentavam dificuldades de vida importantes^(9)^.

Os verdadeiros prejuízos sociais da COVID-19 nas favelas foram invisíveis, uma vez que, no início da pandemia e em grande parte do período, não ocorreu testagem adequada e em larga escala. Além disso, havia subnotificação de grande parcela dos casos, fazendo com que sua real magnitude não fosse revelada nas estatísticas oficiais. Os dados mais próximos dessa realidade foram fornecidos pela Fiocruz, abastecidos por lideranças locais e unidades de Atenção Primária à Saúde (APS)^(10)^.

Além desses problemas que evidenciam a determinação social do processo saúdedoença, a pandemia trouxe angústias pessoais e mudanças bruscas de hábitos de vida, resultando em pânico, medo, dúvidas e incertezas. As pessoas viram a sua condição de liberdade ser confiscada, a desconfiança ser instalada, a capacidade de resposta dos sistemas de saúde entrar em risco e a economia debilitar-se^(11)^.

Essas condições podem ter efeitos na saúde física e mental, por acarretarem mudanças no estilo de vida, tais como a diminuição da prática de atividade física, aumento do consumo de bebidas alcoólicas, cigarros, drogas e de alimentos não saudáveis, além do aumento de estresse e ansiedade^(12)^.

Considerando os impactos psicossociais da pandemia, optou-se por investigar as percepções de um grupo social sobre a COVID-19, com base na Teoria das Representações Sociais (TRS). As representações sociais são consideradas um tipo específico de pensamento social, constituído em conjuntos dinâmicos e direcionado à interpretação do real. Elas determinam a comunicação, compreensão e domínio do meio ambiente, não apenas social, mas também material e ideal. A TRS está principalmente relacionada com o estudo das simbologias sociais e de como esses símbolos influenciam a construção do conhecimento compartilhado e a cultura^(13)^.

Ao definir a TRS como orientadora deste estudo, adota-se o postulado de que toda realidade social é representada e apropriada individual ou coletivamente, sendo reconstituída pelo sistema cognitivo e integrada no sistema de valores de cada sujeito, que depende da sua história e do contexto social e ideológico no qual está inserido^(14)^.

## OBJETIVOS

Analisar a representação social de moradores de uma comunidade vulnerável do Rio de Janeiro sobre a COVID-19, com vistas a fornecer subsídios para as práticas de cuidado de saúde e de enfermagem.

## MÉTODOS

### Aspectos éticos

O Comitê de Ética em Pesquisa da UERJ aprovou este projeto. Respeitando as questões éticas de pesquisa, foi assegurado o direito ao sigilo e anonimato e à desistência em qualquer momento da pesquisa; além disso, foi explicitada a ausência de ônus ou bônus. Os possíveis riscos desta pesquisa relacionaram-se a desconfortos de ordem psicológica que porventura ocorreram durante a coleta de dados. Após a aceitação em integrar a pesquisa, foi oferecido um Termo de Consentimento Livre e Esclarecido (TCLE) em duas vias para assinatura de cada participante, sendo uma para ele e outra para o pesquisador.

### Tipo de estudo e referencial teórico-metodológico

Trata-se de estudo descritivo, com abordagem qualiquantitativa, considerando que as duas técnicas utilizadas no tratamento e análise dos dados são relacionadas à estatística descritiva. Complementarmente, é feita análise qualitativa inferencial na interpretação dos significados das palavras evocadas e na significação dessas palavras na estrutura representacional. O estudo apoiouse na TRS^(14)^, utilizando a abordagem estrutural, a qual pressupõe que a representação é composta por um sistema central e por um sistema periférico, complementados por elementos de contraste^(15)^.

Este estudo está vinculado ao projeto de pesquisa “A construção social do coronavírus e da COVID-19 e suas lições para as práticas de cuidado pessoal, profissional e social”, financiado pelo CNPQ Proc. 422312/2021-5 e pela FAPERJ proc. E-26/211.849/2021.

### Cenário do estudo

O cenário do estudo foi a Comunidade da Rocinha, situada no bairro de São Conrado, no município do Rio de Janeiro (RJ). Ela é caraterizada por alta densidade populacional, habitações precárias, fornecimento irregular de água, coleta de lixo deficiente, esgoto a céu aberto e insuficiente oferta de serviços públicos de forma geral, além do convívio cotidiano com a violência, desigualdade e exclusão, o que permite classificá-la como uma comunidade com alta vulnerabilidade social. Durante a pandemia, medidas de distanciamento social não foram cumpridas, pois, na comunidade de modo geral, ocorreu impossibilidade de exercer trabalho remoto, perda de emprego e de fontes de renda, aglomerações nas próprias casas, além de espaço territorial pequeno e pouco arejado. Essa situação impediu que parte significativa desse contingente populacional se beneficiasse de ações protetivas, o que aumentou o risco de exposição ao contágio pelo coronavírus. Portanto, os recursos básicos necessários às medidas de prevenção do contágio pelo coronavírus não estavam igualitariamente disponíveis para a comunidade estudada.

### Fonte de dados

Os participantes do estudo foram 120 pessoas residentes na Comunidade da Rocinha, com idade maior ou igual a 18 anos e com autonomia para participar na coleta de dados. A inserção dos participantes na amostra foi realizada de modo intencional, considerando as primeiras pessoas que aceitaram participar da pesquisa nos dias agendados para a coleta de dados. O convite foi realizado aos usuários de um serviço de Atenção Básica localizado na comunidade participante.

### Coleta, organização e análise dos dados

Para a coleta dos dados sociodemográficos e clínicos, foi aplicado um formulário contendo variáveis relativas às características sociais dos participantes e à ocorrência da COVID-19. Também foi empregada a técnica de evocações livres de palavras, sendo “COVID-19” o termo indutor, sem justificativa posterior da evocação pelos sujeitos. Essa decisão implicou que as interpretações dos significados expressos pelas palavras foram feitas pelos pesquisadores, apoiando-se em uma dinâmica de júri. Tal dinâmica permitiu que a interpretação dos significados das palavras evocadas na estrutura representacional fosse realizada com base na concordância de três pesquisadores participantes da pesquisa para a interpretação de palavras presentes nos quadrantes. Foram realizadas reuniões da equipe de pesquisa para discussão dos quadrantes já construídos. Em caso de discordância quanto ao significado de palavras específicas, estas eram encaminhadas a três pesquisadores, acatando-se a concordância de dois pesquisadores para a adoção do significado atribuído.

A coleta de dados foi realizada de julho a novembro de 2021, 15 meses após o início da pandemia de COVID-19 no Brasil, que teve o primeiro caso confirmado em 26 de fevereiro de 2020, quando a vacinação já tinha sido iniciada. A coleta se deu individualmente, por meio de um formulário preenchido pelo entrevistador, com base na verbalização de cinco palavras ou expressões que viessem à mente do entrevistado após a enunciação do termo indutor pelo entrevistador.

Os dados sociodemográficos e clínicos foram analisados por meio da estatística descritiva com o emprego do *software Statistical Package for the Social Sciences* (SPSS), que forneceu as frequências simples e relativa das variáveis. Para o tratamento dos dados coletados mediante a técnica de evocações livres, foi utilizado o software EVOC - *Ensemble de Programmes Permettant L’Analyse des* É*vocations*^(16)^, que permite a construção do Quadro de Quatro Casas. Esse quadro é construído com o uso de dois indicadores de importância: a frequência de aparição e a ordem de evocação de cada palavra. Eles são expressos respectivamente pela frequência média do conjunto de palavras e pela média ponderada das ordens de evocação de cada palavra. Tal quadro sintetiza a estrutura e os conteúdos representacionais distribuídos em quatro quadrantes. O cruzamento desses critérios determina os limites de cada quadrante, possibilitando o levantamento tanto das palavras que mais provavelmente pertencem ao núcleo central, por seu caráter prototípico, quanto dos elementos periféricos e de contraste^(15)^.

Considera-se que as palavras com maiores frequências e mais prontamente evocadas, localizadas no quadrante superior esquerdo, são os prováveis elementos que constituem o núcleo central da representação social. Esses elementos são estáveis, não variam em função do contexto imediato e atribuem o significado principal à representação, da qual eles exercem as funções de organização e de estabilização. Nem todos os elementos desse quadrante são centrais, porém o núcleo central da representação encontra-se nesse quadrante, exigindo a associação de técnicas para a sua confirmação. As palavras com maiores ou menores frequências e menos prontamente evocadas, localizadas nos quadrantes superior e inferior direito, constituem o sistema periférico. Esses elementos têm a função de modelar e regular o núcleo central, sustentando a heterogeneidade de posicionamentos apresentada pelo grupo, além de atuar para impedir mudanças no núcleo central e estabelecer relações com as práticas sociais. No quarto quadrante, inferior esquerdo, situa-se a zona de contraste, contendo elementos prontamente evocados, mas que apresentam frequências mais baixas do que a média. Essa zona pode revelar a existência de subgrupos com núcleo central distinto do grupo geral ou somente elementos indicativos de dimensões já reveladas em outros quadrantes, portanto complementares aos demais^(15)^.

Para tornar a análise estrutural mais robusta e confirmar a centralidade dos elementos, uma segunda técnica foi utilizada, a análise de similitude. Ela averigua a quantidade de conexões que um elemento mantém com os outros elementos evocados, expressando tais ligações por meio de índices de similitude, o que resulta em um grafo de similitude. Esse grafo sintetiza graficamente o conjunto das conexões existentes entre os elementos evocados pelo grupo^(17,18)^. Com isso, buscou-se compreender a associação entre os elementos que compõem a representação social da COVID-19 como uma segunda indicação de centralidade dos elementos apontados pela análise prototípica.

## RESULTADOS

Dos 120 participantes, 74 (61,67%) são do sexo feminino, com idades entre 18 e 76 anos, sendo 30 e 39 anos (38,33%) o intervalo que obteve maior percentual. No tocante à escolaridade, a maioria tem ensino médio completo (43; 35,8%). Quanto aos aspectos espirituais, a maioria dos participantes (49; 40,8%) são católicos. Entre os campos de atuação, o principal foi indústria e comércio (50; 41,7%); a renda variou de R$ 1.001,00 a R$ 2.000,00 entre 71 (59,2%) participantes, representando 0,91 a 1,8 salários mínimos à época (R$ 1.100,00); a maioria referiu não ter orientação política (101; 84,2%); e 63 (52,5%) informaram ter sido acometidos pela COVID-19.

Na análise prototípica das evocações livres de palavras, foram identificadas 596 palavras citadas, sendo 117 palavras diferentes. Quanto ao processamento do conteúdo, estabeleceu-se a frequência mínima de sete palavras, conforme Lei de Zipf^(16)^, havendo a exclusão dos termos evocados abaixo dessa frequência; calculou-se a frequência média de evocação (FME) de 18; e a média das ordens médias de evocação (OME) correspondeu a 3,00.

Os resultados da análise prototípica serão descritos e analisados segundo os pressupostos da TRS, em sua abordagem estrutural (Quadro 1).

**Quadro 1:** Quadro de Quatro Casas das evocações ao termo indutor “COVID-19” para pessoas que residem em uma comunidade vulnerável do Rio de Janeiro, Rio de Janeiro, Brasil, 2022 (N = 120)

OME < 3,00				OME ≥ 3,00		
FME	Termo evocado	F	OME	Termo evocado	f	OME
≥ 18	***morte*** ***medo*** perda **doença** sofrimento	70 57 21 18 18	1,900 2,456 3,000 2,536 2,833	tristeza	37	3,297
< 18	desespero dor ***isolamento*** **prevenção** *fome* ***hospital*** **vacina** *pobreza*	17 11 11 10 9 9 9 7	2,941 2,909 3,000 2,900 2,333 2,778 2,667 2,571	ansiedade **cura** ***máscara*** *desemprego* pavor saudade **cuidados** preocupação	15 15 12 11 8 8 7 7	3,467 4,000 3,333 3,182 4,125 4,125 3,143 4,429

***Dimensão informativa ou conceitual*** *; **dimensão do campo de representação ou imagem**; dimensão social; dimensão afetivo-atitudinal.*

De acordo com o exposto no Quadro 1, no quadrante superior esquerdo dessa análise, relativo ao núcleo central, observa-se uma orientação afetivo-atitudinal negativa ante a COVID-19, presente nos termos “medo”, “perda” e “sofrimento; juntamente, há uma dimensão conceitual relativa às informações incorporadas sobre a COVID-19, expressas nos termos “morte” e “doença”. O termo mais evocado pelos participantes foi “morte”, apresentando frequência 70, sendo a mais elevada de todos os quadrantes que compõem o Quadro de Quatro Casas, mas foi a palavra que apresentou a menor ordem média de evocação (OME), indicando ser o elemento mais prontamente evocado. O segundo termo mais evocado foi “medo”, com frequência 57; a palavra “perda” teve a terceira maior presença entre os participantes, 21. Os elementos “doença” e “sofrimento” tiveram as menores frequências entre as evocações do provável núcleo central da representação social.

Nos quadrantes superior e inferior direito, constituintes do sistema periférico, a palavra “tristeza” obteve frequência 37, sendo o único componente da primeira periferia. Na segunda periferia os componentes foram “ansiedade”, “cura”, “máscara”, “desemprego”, “pavor”, “saudade”, “cuidados” e “preocupação”. Os elementos de destaque da periferia da representação foram “ansiedade” e “cura”, pelas suas frequências elevadas dentro do quadrante^(15)^. Com exceção dos termos “cura”, “cuidados” e “máscara”, os demais apresentaram conotação negativa, reforçando assim a atitude geral negativa diante da COVID-19. Já os termos “cuidados” e “preocupação” foram os que tiveram menor frequência no quadrante.

No quadrante inferior esquerdo, relativo à zona de contraste, estão “desespero”, “dor”, “isolamento”, “fome”, “hospital”, “vacina” e “pobreza”, que revelam dimensões já presentes em outros quadrantes. Eles exercem uma função complementar ao núcleo central, reforçando os seus significados em apontar uma representação alternativa. No entanto, a dimensão social tem maior destaque nesse quadrante, quando comparado aos demais, expressando-se por intermédio das palavras “fome” e “pobreza”.

Pode-se observar um conjunto de palavras e seus significados que apresentaram maiores frequências e foram prontamente evocados (menores ordens de evocação), composto por “morte” e “medo”. Elas são acompanhadas do significado expresso por “sofrimento” e “tristeza”, que, somados, representam a terceira maior frequência do quadrante. Pode-se considerar que a representação social da COVID-19, para os moradores da Rocinha, organiza-se ao redor do significado do conjunto “morte-medo-sofrimento” (Figura 1).

**Figura 1:** Grafo de similitude das evocações livres ao termo indutor “COVID-19”, Rio de Janeiro, Rio de Janeiro, Brasil, 2022 (n = 115)

A análise de similitude possibilita a visualização gráfica da rede de ligações mais fortes estabelecidas entre os elementos do Quadro de Quatro Casas. Permite a corroboração da centralidade dos termos da representação social da COVID-19 e a expressão dos conteúdos representacionais mais interligados (Figura 1).

O grafo de similitude apresenta três núcleos de sentido principais (ou nós), caracterizados por palavras que apresentam os maiores números de conexões. Esses núcleos são expressos pelos termos “morte”, com 12 ligações; “medo”, com 7 ligações; e “tristeza”, com 7 ligações. Observa-se a função de organização exercida pela palavra “morte” sobre os demais elementos, tanto pelo número de ligações quanto pela força dessas ligações com as evocações agregadas: “medo (0,29)”, “tristeza (0,20)”, “doença (0,15)”, “perda (0,15)”, “desespero (0,14)”, “sofrimento (0,10)”, “ansiedade (0,08)”, “vacina (0,07)”, “saudade (0,07)”, “pavor (0,06)”, “hospital (0,06)” e “dor (0,06)”. O termo “medo” se configura como segundo elemento em número de ligações (sete elementos associados) e conectouse com “morte” (0,29), “tristeza” (0,16), “hospital” (0,08), “perda” (0,07), “ansiedade” (0,07), “sofrimento” (0,06) e “pobreza” (0,06). Os dois elementos citados mostraram-se como centrais na análise prototípica (Figura 1) e confirmaram essa centralidade na análise de similitude.

O termo “tristeza” teve sete conexões e valores semelhantes de conexões com “morte” (0,20), “medo” (0,16), “sofrimento” (0,09), “perda” (0,09), “dor” (0,08), “doença” (0,06) e “cura” (0,06). Por um lado, essa configuração indica a possibilidade de ser este um elemento central, apesar da sua localização na primeira periferia do Quadro de Quatro Casas. Por outro lado, reforça a proposição acima enunciada de que esse termo expressa o mesmo significado de “sofrimento” (central na análise prototípica). Os demais elementos participantes da análise de similitude confirmam as suas posições na análise prototípica.

## DISCUSSÃO

Os resultados apresentados apontam uma realidade compreendida e construída no sistema sociocognitivo do grupo de participantes pertencentes a comunidades vulneráveis do Rio de Janeiro, com base em valores, vivências e contexto social e cultural no qual estão inseridos.

Moscovici^(19)^ propõe que a organização de uma representação social se dá em três dimensões: a dimensão informativa ou conceitual, o campo de representação ou imagem e a dimensão afetivoatitudinal ou relativa aos julgamentos. Essas dimensões são descritas pelo autor como:

A informação corresponde à organização do conhecimento de um determinado grupo acerca de um objeto social, variando de acordo com a quantidade e a qualidade desse conhecimento. O campo de representação remete à ideia de imagem, de modelo social, ao conteúdo concreto e limitado das proposições atinentes a um aspecto preciso do objeto das representações. Refere-se à organização dos elementos já estruturados na representação. Por fim, a atitude, a mais frequente das três dimensões, refere-se à orientação do comportamento adotado acerca do objeto da representação social, sendo ela que dinamiza e orienta decisivamente as condutas relacionadas ao objeto representado, provocando um conjunto de reações emocionais e comprometendo as pessoas com maior ou menor intensidade^(19)^.

Partindo desses pressupostos, ao analisar o Quadro 1, observa-se que o possível núcleo central da representação social é composto por significados que associam a COVID-19 à morte, ao medo, à doença, às perdas e ao sofrimento. Esses conteúdos simbólicos expressam a experiência e a conscientização dos participantes em relação à gravidade da doença, mostrando ainda os sentimentos negativos vivenciados, ligados à insegurança e à angústia perante a alta mortalidade e gravidade da doença. Dessa forma, pode-se afirmar que o núcleo central é caracterizado por alguns elementos normativos, morte e medo. Em outras palavras, são elementos que foram ativados com base na avaliação ou no julgamento que esse grupo desenvolveu sobre a COVID-19 e que orientaram a construção psicossocial dessa doença. Além disso, esse núcleo central é constituído por elementos funcionais (perdas, doença e sofrimento) associados às experiências vivenciadas durante a pandemia.

A relação entre os termos do núcleo central sugere que a COVID-19 é uma doença que leva à morte, sendo esta, ao mesmo tempo, uma dimensão conceitual e imagética da representação, que desperta sentimentos de sofrimento, perda e medo, constituindo uma dimensão afetivo-atitudinal de caráter negativo.

A respeito da dimensão afetiva, que alguns autores denominam de “dimensão atributiva”, os afetos são “a coloração emotiva que impregna a existência humana e, em particular, a relação com o mundo”^(20)^. Ela abarca os sentimentos e as emoções contidas nas cognições ou comportamentos de julgamento, ativados pela avaliação do objeto social, revelando-se neste estudo francamente negativa, o que demonstra o pânico despertado por uma transmissível e letal doença desconhecida.

Esses elementos são fundamentados na alta taxa de mortalidade que a pandemia apresentou no Brasil até o primeiro semestre de 2022. A morte é uma situação difícil e assustadora, cuja alta frequência e velocidade com que ocorreu durante a pandemia tiveram um considerável impacto sobre as construções psicossociais do grupo estudado — construções expressas em conteúdos representacionais afetivos negativos. O termo “morte” apresentou a maior frequência, foi o mais prontamente evocado e teve o maior número de conexões com outros elementos, indicando ser um elemento estruturante da representação da COVID-19. As associações se deram com os léxicos que exprimem conteúdos afetivos como medo, tristeza, perda, desespero, sofrimento, ansiedade, saudade, pavor e dor. Ligou-se apenas tangencialmente a conteúdos atrelados a uma percepção clínico-biomédica da pandemia, como doença, hospital e vacina, revelando importante relação da morte com constituintes psicológicos e emocionais.

O medo estabeleceu importante relação com a morte, fazendo conexões com vocábulos também ligados a constituintes psicológicos e emocionais, como “tristeza”, “perda”, “ansiedade”, “sofrimento”, além de “hospital” e “pobreza”, mostrando-se também como elemento estruturante da representação. Entende-se que o medo pode estar atrelado à possibilidade real e imediata da infecção pelo coronavírus, que leva à doença e possivelmente à morte, trazendo à tona sua iminência real e simbólica. A dimensão expressa pelo medo também contempla vivências associadas às lacunas de conhecimento sobre a COVID-19, no que se refere ao modo de transmissão, à fisiopatologia da doença e à vulnerabilidade das pessoas quanto à exposição, de modo que são assumidos riscos consideráveis e temerários, provocando temor e apreensão^(21)^.

Os cognemas “perda” e “sofrimento” revelam dimensões psicoemocionais ligadas à morte, estando relacionados à finitude da vida ou à perda da vivência com amigos, familiares e entes queridos.

Os elementos centrais têm a função de organizar e dar estabilidade à representação de determinado objeto social, conferindo significado a ele. O conteúdo do núcleo central é composto por elementos estáveis, que não variam em função do contexto imediato. Esse núcleo é determinado pela natureza do objeto representado, pelo tipo de relações que o grupo mantém com esse objeto e pelo sistema de valores e normas sociais que constituem o meio ambiente ideológico do momento e do grupo^(15)^.

No sistema periférico, o termo “tristeza” apresenta forte conexão com os léxicos “morte” e “medo”, expressando uma dimensão afetivo-atitudinal da representação social da COVID-19. Essa palavra, de orientação negativa, foi ativada pelo objeto social analisado^(22)^. Ela associa-se às experiências vivenciadas, adquiridas, elaboradas e ressignificadas descritas nos elementos centrais “morte”, “perda” e “sofrimento”. Pode, igualmente, estar associada à metáfora da morte social, já que a COVID-19 impôs o afastamento dos indivíduos da rede de apoio social, bem como alterou os papéis desempenhados na sociedade. Essa dimensão representacional demonstra as vivências afetivoemocionais às quais os participantes foram submetidos, gerando estresse e sofrimento^(23)^.

Ainda no sistema periférico, a segunda periferia da representação social da COVID-19 retrata não só elementos normativos, mas também estampa elementos de caráter funcional. Além disso, exprime as dimensões conceitual e afetiva, além de trazer uma dimensão nova no sistema periférico, a dimensão social da doença, expressa na palavra “desemprego”. Os elementos que figuram na segunda periferia sugerem uma articulação com o provável sistema central identificado, sobretudo o termo “saudade” associado à ideia de morte e perda, bem como ansiedade, pavor e preocupação, relacionados ao medo e ao sofrimento despertados pela COVID-19. Ambos os termos parecem ser coerentes com o termo “tristeza”, posicionado na primeira periferia da representação. Configura-se, portanto, mais uma vez, como elementos de proteção ao núcleo central e vislumbra o predomínio de sentimentos negativos.

Os elementos periféricos têm a função de modelar e regular o núcleo central, sustentando a heterogeneidade de posicionamentos apresentada pelo grupo; assim, percebe-se a coerência existente entre o núcleo central e a periferia nesta análise^(15)^.

As palavras “cura”, “máscara” e “cuidados” sugerem a correspondência com o constituinte central “doença”. Nessa condição, manifesta uma dimensão conceitual pertencente ao universo reificado do coronavírus e da COVID-19. Evidenciando os elementos funcionais da representação, esses cognemas estabelecem uma relação com as ações adotadas pelo grupo para o enfrentamento da doença e da morte, colocando-se como estratégias para evitar o acometimento pelo vírus. Achados semelhantes foram relatados em pesquisa desenvolvida na Região Norte do Brasil, que indicou a utilização de álcool, sabão e máscaras bem como o isolamento enquanto fatores de prevenção e de proteção diante da propagação do vírus^(23)^.

O elemento “desemprego” não tem correspondência no núcleo central nem na primeira periferia, apontando para uma nova dimensão dos impactos sociais da pandemia presente nessa representação. Tal dimensão é observada de forma mais destacada na zona de contraste da análise prototípica, nos elementos fome e pobreza. Esses termos demonstram que a COVID-19 acentuou as diferenças sociais, agravou o número de desempregados, determinou a redução dos salários e aumentou a exclusão social. Eles evidenciam o impacto econômico que essa doença causou na sociedade, principalmente nas classes sociais mais vulneráveis, desvelando a faceta cruel desse mal que acometeu para além dos sintomas físicos e psíquicos. Adicionalmente, demonstra a vulnerabilidade do grupo estudado advinda da desigualdade social existente antes da pandemia.

Pesquisas apontam o impacto da pandemia não só na saúde pública, mas também no aumento do desemprego e influência sobre o mercado de trabalho, principalmente entre a população jovem^(24,25)^. Com esse evento, as famílias que se encontravam em contexto de vulnerabilidade social passaram a apresentar expressiva suscetibilidade em razão do desemprego acelerado, vínculo de trabalho precário ou informal e consequente queda da renda, além dos locais de moradia vulneráveis e insalubres, havendo a necessidade de obter recursos de programas sociais de curto prazo^(26)^. Além dos impactos pragmáticos, o desemprego desencadeia o desarranjo e o afastamento identitário do sujeito, com consequências para a saúde mental e identidade social em um momento de fragilidade extrema^(27)^.

Os demais elementos constituintes da zona de contraste reafirmam as percepções apresentadas nos demais quadrantes. Trazem elementos normativos e funcionais, tendo a dimensão afetiva representada pelos vocábulos “desespero” e “dor”, relacionados com os elementos “morte”, “perda” e “sofrimento” elencados no núcleo central; “tristeza” na primeira periferia e “pavor, saudade e ansiedade” descritos na segunda periferia. Tais elementos revelam de forma inevitável a representação atravessada por elementos de natureza negativa.

Já os termos “isolamento”, “prevenção”, “hospital” e “vacina” referem-se aos elementos “cura”, “máscara” e “cuidados”, refletindo medidas para evitar e enfrentar a doença e uma possível morte. Esses elementos elucidam o universo reificado pautado nos saberes científicos, o qual demonstra a familiaridade que os participantes têm com as medidas necessárias para evitar a contaminação e seu agravamento, caracteriza-se como dimensão informativa (conceitual) e enumera os elementos funcionais da representação da COVID-19.

O termo “vacina” mostrou-se conectado ao léxico “morte” no grafo de similitude, apresentando-se como uma resposta de contraposição à finitude da vida e uma medida de proteção para evitar a doença e a morte. Observa-se a referência ao hospital como um lócus associado à morte e ao medo, demonstrando que essa elaboração simbólica se organizou, em grande parte, devido às imagens veiculadas na mídia retratando as unidades de saúde como cenários de guerra e como campos de batalha e de morte.

Os resultados analisados mostram que a construção psicossocial da COVID-19 pelo grupo foi pautada em construções que a caracterizam como uma doença que destrói, mata e que gera perdas e sofrimento, sendo “morte”, “medo” e “sofrimento” os constituintes estruturantes, o núcleo duro dessa representação. No entanto, apesar de o núcleo central trazer elementos negativos, também se percebe elementos mais neutros relacionados ao enfrentamento da doença, às medidas de prevenção e às práticas para evitar o adoecimento, como formas de resiliência em um cenário de destruição.

A COVID-19 trouxe grandes desafios para a saúde pública, superlotando hospitais e instituições de saúde e trazendo implicações para diversas forças de trabalho^(28)^. Diante dos esforços para combater a pandemia, algumas atividades deixaram de ter espaço no cotidiano do enfermeiro, entre as quais está a educação em saúde. Esta se caracteriza por ser um conjunto de práticas pedagógicas de caráter participativo e emancipatório, com objetivo de sensibilizar, conscientizar e mobilizar para o enfrentamento de situações individuais e coletivas que interferem na qualidade de vida^(29)^.

A sobrecarga de trabalho, resultante do impacto imediato da COVID-19 nos serviços de saúde, resultou em redução das atividades educativas, abrindo brechas para que falsas informações e *fake news* fossem veiculadas nas mídias sociais. Isso modificou elementos simbólicos já consolidados nos grupos sociais, como a adesão à imunização mediante o Programa Nacional de Vacinação.

O desafio dos enfermeiros foi o de realizar essa atividade com soluções criativas, pois a educação em saúde é o meio pelo qual a informação confiável se concretiza e pode informar práticas sociais de autoproteção e de proteção do outro^(29)^. É uma atividade fundamental de cuidado que permite trazer reflexões e quebrar paradigmas, com possibilidade de provocar mudanças nas representações e nas práticas ligadas à COVID-19.

Os elementos representacionais apresentados nesta pesquisa mostram o sofrimento emocional enfrentado pelas pessoas que vivenciaram a pandemia. Esse evento gerou prognósticos sombrios, sentimentos de desamparo social e sofrimento emocional; expôs os mais vulneráveis ao risco aumentado de desenvolver sintomas psicológicos, podendo levar a quadros psicopatológicos. Em tais circunstâncias, constata-se a importância da educação em saúde. Ela destina-se à população em geral e deve ser considerada uma atividade-chave no planejamento de ações nas pandemias, visto que interfere no modo de vida e nos comportamentos desenvolvidos diante da realidade vivenciada^(30)^.

Nesse sentido, considerando que a APS é o cenário para condução de práticas resolutivas e responsável pelo cuidado, é preciso que as práticas de enfermagem visem às demandas do ser biopsicossocial e ultrapassem aquelas relativas à saúde física e ao corpo biológico. Entende-se que o acolhimento, a escuta atenciosa e o conforto dos pacientes são estratégias de cuidado complexas que alicerçam o cuidado em saúde. Similarmente, os recursos de matriciamento, visitas domiciliares, grupos terapêuticos e o serviço de telessaúde potencializam a relação com o cuidado e manejo da saúde, podendo reduzir o sofrimento em situações pandêmicas.

A representação social da COVID-19 permitiu desnudar o impacto da desigualdade social na saúde, a vulnerabilidade econômica e a pobreza vivenciada pelo grupo, levando a rupturas sociais ainda maiores do que as já existentes antes da pandemia. As medidas impostas pela pandemia, individuais ou coletivas, para os grupos mais vulneráveis comprometeram as condições de provisão mínima para garantir o sustento cotidiano, a qualidade de vida e a prevenção de doenças, acarretando condições de saúde e de vida ainda mais precárias. Tal cenário impôs restrições ao cuidado integral e ao setor saúde, que teve dificuldades de encontrar soluções para amenizar os efeitos da pandemia. Esse setor conta com a firme presença do Estado na implementação e aprimoramento das políticas de transferência de renda e fortalecimento da saúde pública por meio do Sistema Único de Saúde (SUS).

### Limitações do estudo

Aponta-se como limitação do presente estudo o quantitativo de sujeitos, que impediu a análise comparativa entre aqueles que referiram ter desenvolvido COVID-19 e os que não se infectaram. A comparação teria permitido a discussão das variações na construção social da doença em função do maior ou menor contato com a doença.

### Contribuições para a área da enfermagem

Este estudo permitiu a compreensão da realidade psicossocial vivenciada durante a pandemia de COVID19. Contribuiu para compreender o momento vivido dos atores sociais pertencentes a grupos sociais em situação de vulnerabilidade social e trouxe subsídios para apoiar a implementação do cuidado no contexto pandêmico.

## CONSIDERAÇÕES FINAIS

O estudo revelou que a representação social da COVID-19 se caracteriza pela morte, pelo medo e pela tristeza como seus elementos estruturantes. A constituição dos conteúdos e a organização interna da representação social da COVID-19 fornecem um panorama coerente entre os possíveis elementos centrais e os periféricos. Isso possibilita entender como o grupo elaborou e se posicionou negativamente diante da COVID-19, enquanto doença letal, marcada pelos sentimentos de medo, sofrimento, tristeza e saudade. Este trabalho também expôs elementos funcionais que traduzem as práticas do grupo, relacionadas à prevenção para o enfrentamento da doença, como o uso de máscara, o isolamento e a vacina. Além disso, evidenciou a pobreza e a fome como produtos sociais e econômicos da pandemia, que se expressaram de forma predominante em grupos sociais já vulneráveis, como os moradores de favelas.

A análise da representação social da COVID-19 permitiu uma reflexão sobre a necessidade de aperfeiçoamento dos cuidados de saúde prestados pelos enfermeiros e profissionais de saúde. Mostrou que é preciso mudar a configuração da assistência prestada a essa população, considerando não só os efeitos físicos da doença, mas também aqueles psicossociais.
